# CBFB Break-Apart FISH Testing: An Analysis of 1629 AML Cases with a Focus on Atypical Findings and Their Implications in Clinical Diagnosis and Management

**DOI:** 10.3390/cancers13215354

**Published:** 2021-10-26

**Authors:** Richard K. Yang, Gokce A. Toruner, Wei Wang, Hong Fang, Ghayas C. Issa, Lulu Wang, Andrés E. Quesada, Beenu Thakral, Keyur P. Patel, Guang Peng, Shujuan Liu, C. Cameron Yin, Gautam Borthakur, Zhenya Tang, Sa A. Wang, Roberto N. Miranda, Joseph D. Khoury, L. Jeffrey Medeiros, Guilin Tang

**Affiliations:** 1 Department of Hematopathology, The University of Texas MD Anderson Cancer Center, Houston, TX 77030, USA; rkyang@mdanderson.org (R.K.Y.); gatoruner@mdanderson.org (G.A.T.); wwang13@mdanderson.org (W.W.); HFang@mdanderson.org (H.F.); AQuesada@mdanderson.org (A.E.Q.); bthakral@mdanderson.org (B.T.); kppatel@mdanderson.org (K.P.P.); cyin@mdanderson.org (C.C.Y.); swang5@mdanderson.org (S.A.W.); roberto.miranda@mdanderson.org (R.N.M.); JKhoury@mdanderson.org (J.D.K.); ljmedeiros@mdanderson.org (L.J.M.); gtang@mdanderson.org (G.T.); 2 Department of Leukemia, The University of Texas MD Anderson Cancer Center, Houston, TX 77030, USA; GCIssa@mdanderson.org (G.C.I.); gborthak@mdanderson.org (G.B.); 3 Department of Clinical Cancer Prevention, The University of Texas MD Anderson Cancer Center, Houston, TX 77030, USA; lwang14@mdanderson.org (L.W.); gpeng@mdanderson.org (G.P.); 4 Parkview Regional Medical Center, Allied Hospital Pathologists, Fort Wayne, IN 46845, USA; sjliu02@gmail.com

**Keywords:** FISH, *CBFB rearrangement*, *CBFB-MYH11*, RT-PCR, atypical findings, additional chromosome16 aberrations (AC16As), next-generation sequencing (NGS)

## Abstract

**Simple Summary:**

The inv(16)/t(16;16) AML is a disease that is considered relatively easy and straightforward to be diagnosed in the clinical laboratories. Up to date, CBFB FISH and/or CBFB-MYH11 RT-PCR are still the major diagnostic assays utilized in the clinical laboratories. However, incidental CBFB FISH findings and their implication in clinical laboratory diagnostics and management, especially in the era of next-generation sequencing (NGS)-based methods, have not been systemically investigated. In this study, we systemically studied over 1600 AML cases tested with CBFB FISH. Over 5% of cases with a confirmed *CBFB* rearrangement were challenging, including those with discrepant FISH and RT-PCR results and/or atypical FISH findings. Meanwhile, atypical FISH findings usually indicate additional chromosome 16 aberrations (AC16As) overlooked by other methods including RT-PCR and almost all NGS-based methods if following the published parameters. The information revealed in this study will be useful for further workup and interpreting atypical CBFB FISH findings and confirmation of inv(16)/t(16;16) AML diagnosis and related treatment, as well as selection of samples to better validate NGS-based new diagnostic methods.

**Abstract:**

Fluorescence in situ hybridization (FISH) is a confirmatory test to establish a diagnosis of inv(16)/t(16;16) AML. However, incidental findings and their clinical diagnostic implication have not been systemically studied. We studied 1629 CBFB FISH cases performed in our institution, 262 (16.1%), 1234 (75.7%), and 133 (8.2%) were reported as positive, normal, and abnormal, respectively. The last included *CBFB* copy number changes (*n* = 120) and atypical findings such as 3′CBFB deletion (*n* = 11), 5′CBFB deletion (*n* = 1), and 5′*CBFB* gain (*n* = 1). Correlating with *CBFB-MYH11* RT-PCR results, totally 271 *CBFB* rearrangement cases were identified, including five with discrepancies between FISH and RT-PCR due to new partner genes (*n* = 3), insertion (*n* = 1), or rare *CBFB-MYH11* variant (*n* = 1) and eight with *3′CBFB* deletion. All cases with atypical findings and/or discrepancies presented clinical diagnostic challenges. Correlating FISH signal patterns and karyotypes, additional chromosome 16 aberrations (AC16As) show impacts on the re-definition of a complex karyotype and prognostic prediction. The *CBFB* rearrangement but not all AC16As will be detected by NGS-based methods. Therefore, FISH testing is currently still needed to provide a quick and straightforward confirmatory inv(16)/t(16;16) AML diagnosis and additional information related to clinical management.

## 1. Introduction

Acute myeloid leukemia (AML) with inv(16)(p13.1q22)/t(16;16)(p13.1;q22), *CBFB-MYH11* (heretofore referred to as inv(16)/t(16;16) AML), usually shows monocytic and granulocytic differentiation, is characterized by abnormal eosinophils with large basophilic granules, and it is often associated with favorable overall survival when treated appropriately. The presence of *CBFB-MYH11* rearrangement confirms the diagnosis AML with inv(16)/t(16;16) regardless of blast counts [1]. The prevalence of *CBFB-MYH11* rearrangement is approximately 4% in de novo AML and 11% in secondary AML patients [2,3]. Two assays are widely used for detection of *CBFB-MYH11* rearrangement: DNA-based fluorescence in situ hybridization (FISH) [4,5] and RNA-based reverse transcriptase-polymerase chain reaction (RT-PCR) [6,7]. Due to differences in biology, techniques and feasibility between these two assays, FISH and RT-PCR, have apparent advantages and disadvantages that have been widely reported [8]; thus, both assays are offered simultaneously in many laboratories. For example, FISH may be applied for a fast screen to establish the diagnosis and initiate chemotherapy in a timely fashion, whereas RT-PCR is utilized for quantification of *CBFB-MYH11* transcripts and monitoring of minimal residual disease during follow-up [9].

Based on the probe design, there are two types of FISH test for detecting *CBFB* rearrangement: *CBFB* break-apart FISH test (BAP FISH) and *CBFB-MYH11* dual fusion FISH test (DF FISH). The BAP FISH utilizes DNA fragments targeting the *CBFB*-containing region, and labels *5′CBFB* (centromeric) with spectrum orange (red) and *3′CBFB* (telomeric) with spectrum green. A normal signal pattern should exhibit as two fusion (2F) signals, and a typical positive signal pattern for *CBFB* rearrangement is one red, one green, and one fusion (1R1G1F) signals. Since BAP FISH is designed to detect *CBFB* rearrangement regardless of the partner gene(s), a positive FISH result does not necessarily indicate a *CBFB-MYH11* rearrangement. On the other hand, the DF FISH utilizes both *CBFB*-specific (labelled as red, for example) and *MYH11*-specific (labeled as green) probes. A normal signal pattern should be two red and two green (2R2G) signals, and a typical positive signal pattern for *CBFB-MYH11* rearrangement is one red, one green, and two fusion (1R1G2F) signals. The DF FISH is designed specifically for detecting *CBFB-MYH11* rearrangement and it generally does not detect a *CBFB* rearrangement with a partner gene other than *MYH11*. According to the Mitelman Database of Chromosome Aberrations and Gene Fusions in Cancer (https://mitelmandatabase.isb-cgc.org/mb_search (accessed on 17 September 2021)), *MYH11* is the only identified partner gene for *CBFB* rearrangement in AML. Therefore, BAP FISH and DF FISH are considered equivalent for the purpose of confirmatory diagnosis of inv(16)/t(16;16) AML.

Atypical findings could be encountered during CBFB BAP FISH testing, such as insertion resulting in a false-negative FISH result [10,11], *3′CBFB* deletion [12,13,14,15,16,17], and rare *CBFB-MYH11* isoforms [18,19], and have been reported as case reports. Atypical findings, including atypical signal patterns, could pose diagnostic challenges. For example, a signal pattern of 1R1F or 1G1F by BAP FISH indicates partial deletion of *CBFB* gene and/or its flanking region, which could be due to an interstitial deletion, an unbalanced inversion /translocation, or an insertion. To confirm *CBFB* rearrangement in such cases, an alternative technique (e.g., RT-PCR) is essential. Currently, the prevalence and clinical significance of these atypical findings generally remain unknown in inv(16)/t(16;16) AML.

In this study, we retrospectively analyzed 1629 AML patients with CBFB BAP FISH tests performed in our institute. Atypical findings, including atypical signal patterns (1R1F, 1G1F), discordance results between BAP FISH and RT-PCR, and t(16q22;v) (partner chromosome(s) and/or band level(s) other than 16p13.1), and their relevance for clinical diagnosis and management were systemically studied. Their implications for next-generation sequencing (NGS)-based methods were also explored. 

## 2. Materials and Methods

### 2.1. Cases

We searched the database of the Clinical Cytogenetics Laboratory in the Department of Hematopathology, The University of Texas MD Anderson Cancer Center, for *CBFB* BAP FISH tests performed from 1 June 2000 through 31 May 2021. The clinical, pathologic, and other laboratory information were collected through electronic medical chart review. This study was approved by Institutional Review Board (IRB) of MD Anderson Cancer Center and performed in accordance with the Declaration of Helsinki. 

### 2.2. Karyotype Analysis

As we reported previously [20,21], conventional G-banded chromosomal analysis or karyotyping was performed in bone marrow (BM) aspirate and/or peripheral blood, which were inoculated into cell culture for 24 h and 48 h without mitogens. Routinely, 20 metaphases were analyzed for each specimen and the final results were reported by following the 2020 International System for Human Cytogenetics Nomenclature (ISCN 2020) guidelines [22]. An aberration not appreciated by karyotyping but revealed by other sensitive methods, such as FISH, RT-PCR, and/or array comparative genomic hybridization (aCGH), is considered as a cryptic chromosomal abnormality. A complex karyotype is defined as ≥3 chromosomal abnormalities, of which at least one chromosomal abnormality is structural, including inv(16)(p13q22) or t(16;16))(p13;q22) [22]. 

### 2.3. Fluorescence in Situ Hybridization (FISH) Analysis

**Figure 1:**
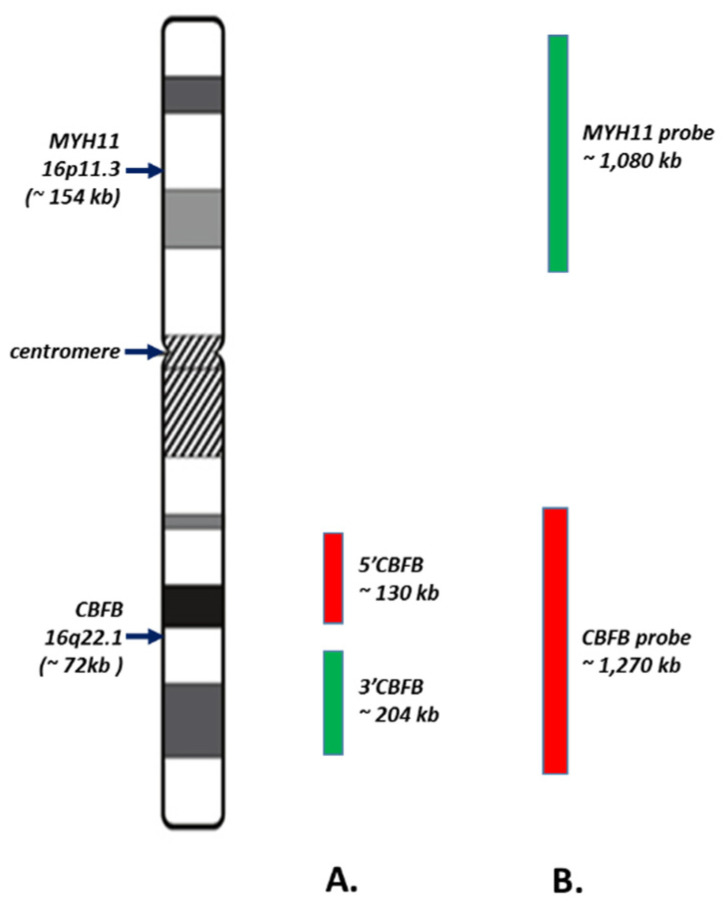
Schematic illustration of CBFB break-apart (BAP) and CBFB-MYH dual fusion (DF) FISH probe sets applied in this study. Information was obtained from the user’s guide provided by the manufacturers. (**A**). CBFB BAP probe set with coverages of *5′CBFB* and flanking region (~130 kb) labeled with red dye, and *3′CBFB* and flanking region (~204 kb) labeled with green dye. (**B**). CBFB-MYH11 DF probe set with coverages of *CBFB* and flanking region (~1270 kb) labeled with red dye, and *MYH11* and flanking region (~1080 kb) labeled with green dye. The sizes are not to scale.

FISH analysis with CBFB Dual Color Break Apart Rearrangement Probe (Abbott Molecular, Des Plaines, IL, USA) was performed in all cases included in this study. The cutoff value for *CBFB* rearrangement established in our lab is 4.2% for a typical signal pattern (1R1G1F). The cutoff value for some atypical signal patterns was also established during validation: *3*′*CBFB* deletion (1R1F) < 0.4%, *5*′*CBFB* deletion (1G1F) < 0.4%, and loss of one copy of *CBFB* (1F) < 5%. The CBFB-MYH11 Dual Fusion Probe (CytoTest Inc., Rockville, MD, USA) was performed on a few challenging cases with atypical signal pattern(s). The information of both probe sets applied in this study is illustrated in Figure 1 with detailed gene coverages. 

### 2.4. CBFB-MYH11 RT-PCR Analysis

A Fluidigm nanofluidics-based Acute Leukemia Translocation Panel (LTP) was performed in newly diagnosed acute leukemia cases [23]. For cases positive for *CBFB-MYH11* fusion by LTP screening and/or with a history of inv(16)/t(16;16), real-time RT-PCR was performed to quantitatively measure and dynamically monitor *CBFB-MYH11* transcript levels. The sensitivity of RT-PCR for *CBFB-MYH11* fusion transcripts is between 1 in 10,000 and 1 in 100,000 [23,24]. 

### 2.5. The aCGH Analysis

As reported previously, aCGH was applied to detect genome-wide copy number aberrations (CNAs) for a subset of new AML cases with high blast counts per the clinician’s request. A custom 4 × 180 K format from Agilent Technologies (Santa Clara, CA, USA) with emphasis on cancer-related genes was developed and validated. The average resolution of this assay for a defined CNV was 25 kb [20]. 

### 2.6. Statistical Analysis

A Chi-Square test was employed for statistical analyses of frequencies in this study, and statistical significance was considered to be present if *p* < 0.05. 

## 3. Results

### 3.1. CBFB BAP FISH Results

A total of 2809 CBFB BAP FISH tests were performed on 1629 patients and formed the study cohort. From this group, 356 (12.7%) tests performed on 262 (16.1%) patients were reported as positive for *CBFB* rearrangement (55 patients tested positive two or more times, and most of them had a relapse) and 150 (5.3%) tests on 133 (8.2%) patients were reported as abnormal, whereas the remaining tests/patients (*n* = 1234, 76%) yielded a normal result. Of the 133 patients with abnormal results, 120 showed copy number changes, including loss of one copy of *CBFB* (*n* = 84), gain of one or more copies of *CBFB* (*n* = 34), and a mixture of subclones with loss or gain of one copy of *CBFB* (*n* = 2). Other abnormal results included 3′CBFB deletion (*n* = 11), 5′*CBFB* deletion (*n* = 1), and gain of 5′CBFB (*n* = 1) (Table 1). 

### 3.2. Correlation between CBFB BAP FISH and CBFB-MYH11 RT-PCR Results

Technically, the PCR-based methods employed are extremely sensitive (e.g., the sensitivity of CBFB-MYH11 RT-PCR is 1/10,000 to 1/100,000) over any FISH-based methods (e.g., the LOD of BAP FISH was 4.2% for a case with typical signal pattern of 1R1G1F in this study). To exclude discordances caused by the sensitivities of both methods used in this study, a positive RT-PCR result with a percentage of *CBFB-MYH11* transcripts below 4.2% was intentionally considered as concordant with the negative BAP FISH result. By following this rule, a total of 974 cases with concurrent BAP FISH and RT-PCR results were analyzed. Of the 262 BAP FISH positive cases, 258 (98.5%) were RT-PCR positive while four (1.5%) were RT-PCR negative. Of the 645 BAP FISH normal cases, one (0.2%) was RT-PCR positive (the percentage of *CBFB-MYH11* to *ABL1* > 100%). Of the 11 cases with 3′*CBFB* deletion, eight (73%) were RT-PCR positive. The other 56 cases with abnormal BAP FISH results all showed negative RT-PCR results (Table 2). Taken together, a total of 271 cases exhibited a *CBFB* rearrangement detected by either BAP FISH and/or RT-PCR in this cohort. 

**Figure 2:**
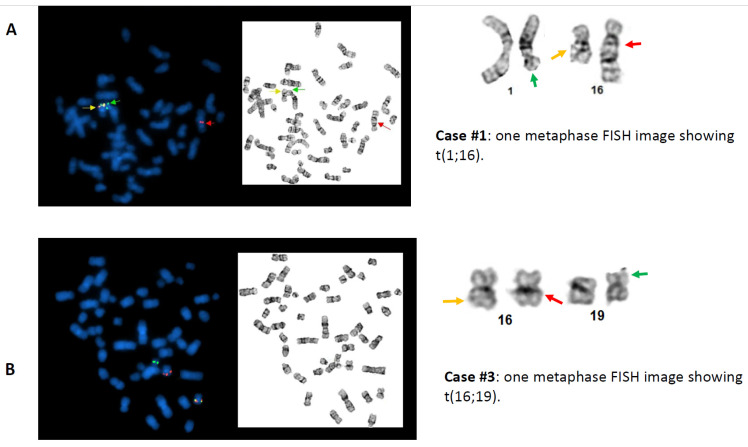
CBFB BAP FISH demonstrated that *3′CBFB* signal (green) was translocated on an abnormal chromosome 1 (**A**). Case #1 and an abnormal chromosome 19 (**B**). Case #3, respectively. From left to right: metaphase FISH image with DAPI; inverted metaphase FISH image to show chromosome morphology; affected chromosomes and their normal homologs. Red: *5′CBFB*; Green: *3′CBFB;* Fusion (yellow)*:* intact *CBFB*.

**Figure 3:**
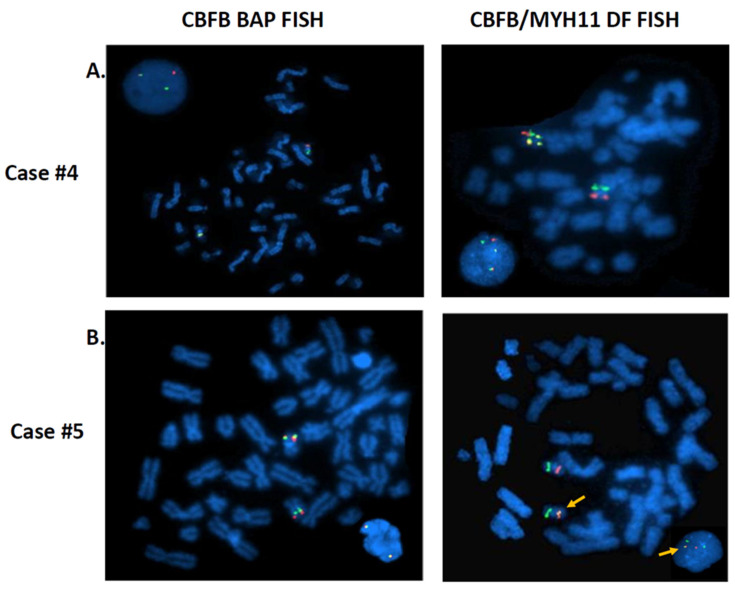
FISH studies with CBFB BAP FISH and CBFB-MYH11 DF FISH in two cases. (**A**). Case #4: Both BAP FISH (left, 1R1G1F signal pattern) and DF FISH (right, 1R1G2F) showed typical signal patterns for *CBFB-MYH11* rearrangement. RT-PCR was negative in this case. (**B**). Case #5 showed a negative CBFB BAP FISH result (left). CBFB-MYH11 DF FISH indicated an insertion of *MYH11* (green) into the *CBFB* (red), forming a fusion signal (right). RT-PCR was positive in this case.

**Figure 4:**
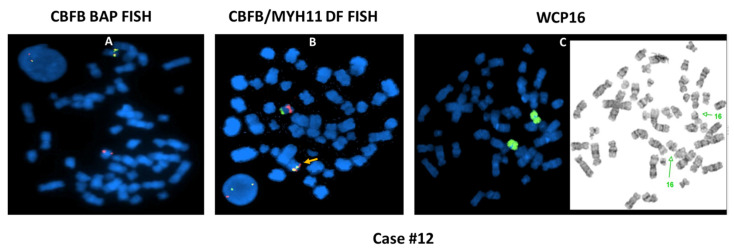
Illustration of cryptic and complicated *CBFB-MYH11* rearrangement in case #12: atypical FISH signal pattern (1R1F, **A**) by BAP FISH; one fusion signal (1R1G1F, **B**) by DF FISH. Whole chromosome 16 painting (wcp16) (**C**) excluded a possible recombination between one chromosome 16 and a non-16 chromosome.

**Figure 5:**
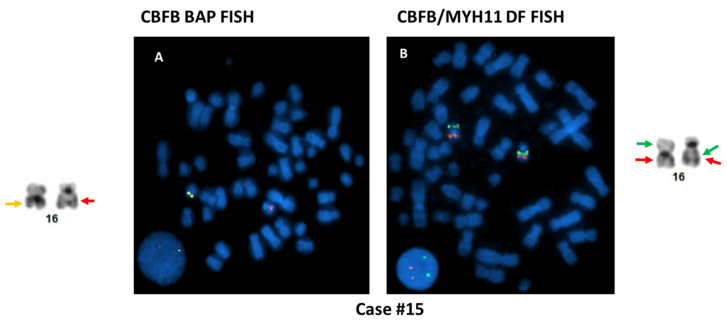
Illustration of case #15 with inv(16) and an additional chromosomal 16 abnormality. An atypical FISH signal pattern (1R1F, **A**) was detected by BAP FISH. However, no *CBFB-MYH11* fusion signal was detected by DF FISH (**B**), though the metaphase image indicated that the *MYH11* was relocated on 16q (RT-PCR was also negative).

To further investigate the possible causes for the discordant BAP FISH and RT-PCR results, CBFB-MYH11 DF FISH was performed in four cases (cases #4, #5, #12, and #15) and aCGH assay was also performed in four cases (cases #4, #5, #9 and #17), as listed in Table 3. The cause of discordant BAP FISH and RT-PCR results in the first three cases (cases #1–#3) was most likely attributable to *CBFB* rearrangement with a partner gene other than *MYH11*. By conventional cytogenetic analyses, these three cases exhibited t(1;16)(q21;q22), t(2;16)(q37;q22), and t(16;19)(q22;q13.3) involving the 16q22 band containing *CBFB,* leading to a typical signal pattern (1R1G1F) for *CBFB* rearrangement by BAP FISH; but RT-PCR for *CBFB-MYH11* was negative. The metaphase FISH images captured during the BAP FISH tests demonstrated that the *5′CBFB* signal (centromeric, R) was retained on the abnormal chromosome 16, whereas the *3′CBFB* signal (telomeric, G) was relocated to the abnormal chromosomes 1 (Figure 2A), 2, and 19 (Figure 2B), respectively, indicating the possibility of *CBFB* rearrangement with novel partner gene(s) other than *MYH11*. Case #4 showed inv(16)(p13;q22) by conventional cytogenetics, and a typical positive signal pattern (1R1G1F) was detected in 67% of cells by BAP FISH. Further investigation with DF FISH also showed a typical signal pattern (1R1G2F) for *CBFB-MYH11* rearrangement (Figure 3A). However, repeated RT-PCR tests were negative, and aCGH was normal, which could be due to a rare/novel *CBFB-MYH11* variant. Case #5 exhibited a normal male karyotype with an inv(9)(p12q13) (a polymorphism present in healthy individuals). The BAP FISH result was negative. However, DF FISH indicated a small segment of *MYH11* (green) that was inserted into the *CBFB* fragment (red), forming a relatively weak fusion signal that could be overlooked (Figure 3B). The RT-PCR was positive, further confirming the *CBFB-MYH11* rearrangement in this case. A concurrent aCGH assay revealed a loss of approximately 140 kb of 16p13.11 (nt 15,815,457–15,954,987) including part of *MYH11.* Eight (cases #6–#13) of 11 cases with *3′CBFB* deletion by BAP FISH were RT-PCR positive, consistent with an unbalanced *CBFB-MYH11* rearrangement: Inversion of affected chromosome 16 was apparent in all eight cases, but deletion was not detected by chromosomal analysis in three cases (cryptic, cases #7, #10, #11). In case #12, the BAP FISH showed 1R1F signal pattern, the DF FISH showed a fusion signal on the “shorter” chromosomes 16, and the whole chromosome 16 painting (WCP16) excluded a possible recombination between one chromosome 16 and another non-16 chromosome (Figure 4), supporting the concurrent events: inversion plus deletion, on the affected chromosome 16. In contrast, cases #14–#16 also showed *3′CBFB* deletion and case #17 showed *5′CBFB* deletion, but they were all RT-PCR negative, which excluded a *CBFB-MYH11* rearrangement. Interestingly, they all showed chromosomal aberration(s) involving *CBFB* gene. For example, the DF FISH showed that *MYH11* was relocated to 16q through a pericentric inversion, likely inv(16)(p13.1q13) at a different band level/breakpoint, but certainly did not form a *CBFB-MYH11* fusion detectable by BAP FISH, DF FISH, and RT-PCR in case #15 (Figure 5). Therefore, a *CBFB* rearrangement but not with *MYH11* cannot be completely excluded in cases #14 to #17. 

### 3.3. Correlation between Karyotype Analysis and CBFB BAP FISH Results

Eight BAP FISH positive cases had insufficient (*n* = 3) or failed (*n* = 5) chromosomal analysis in this cohort. Therefore, we correlated karyotype and BAP FISH results in 263 cases with confirmed *CBFB* rearrangement, including 254 BAP FISH positive, eight BAP FISH abnormal (*3′CBFB* deletion) but RT-PCR positive, and one BAP FISH normal but RT-PCR positive case (Table 4). Most (260/263) of these cases exhibited apparent chromosome 16 abnormalities, including inv(16) (*n* = 240, 91.2%), t(16;16) (*n* = 17; 6.5%), or t(16q22;v) (*n* = 3, 1.1%, cases #1–#3) that were detected by conventional cytogenetic analysis. Three cases (1.1%) exhibited a normal karyotype, suggesting cryptic chromosomal abnormalities leading to *CBFB* rearrangement. Of the 263 patients, 139 (53%) cases had additional chromosomal abnormalities (ACAs) other than those involving chromosome 16, mostly trisomy 22 (*n* = 55, 21%) and/or trisomy 8 (*n* = 53, 20.2%), and 81 (31.2%) had a complex karyotype by standard definition. Nine (3.4%) cases exhibited apparent additional chromosome 16 abnormalities (AC16As) other than the co-existing inv(16), t(16;16) or t(16q22;v). Interestingly, the prevalence of ACAs was higher but did not show statistically significant differences between cases with AC16As and cases without AC16As (7/9 vs. 132/254, *p* = 0.127); however, the prevalence of complex karyotype by standard definition was statistically significantly higher in cases with AC16As than that without AC16As (6/9 vs. 75/254, *p* = 0.017) in this cohort. The presence of complex karyotype is usually considered as an indicator for poor prognosis in all AML cases [2,3].

## 4. Discussion

FISH testing using either a CBFB break-apart probe or a CBFB-MYH11 dual fusion probe set is one of the most commonly used methods to confirm a diagnosis of inv(16)/t(16;16) AML in clinical diagnostic laboratories [5,8]. FISH can be used as a sole test or, more commonly, in combination with conventional cytogenetics and/or RT-PCR. However, these FISH approaches and their importance for clinical diagnostics and management of inv(16)/t(16;16) AML patients have not been systemically assessed. In this study, 271 *CBFB* rearrangement positive cases from 1629 AML patients were identified. To the best of our knowledge, this is the largest cohort of patients with *CBFB* rearrangement in the literature. It is necessary to point out that a CBFB BAP FISH test was performed either per request by a clinician or a hematopathologist for selected AML cases with myelomonocytic or monocytic differentiation only or as a confirmatory test after detecting 16q abnormalities by conventional cytogenetics before 2017 [25]. From 2017, a CBFB BAP FISH has been performed on all newly diagnosed AML patients. This could account for the higher detection rate of *CBFB* rearrangement (16.6%) by FISH in our cohort than the reported 5% of inv(16)/t(16;16) by conventional cytogenetics only in all AML cases [3].

In our study, approximately 5% (13/271) of cases with confirmed *CBFB* rearrangement presented diagnostic challenges, including five (1.8%) cases with discordant FISH and RT-PCR results and eight (3%) cases that exhibited a *3′CBFB* deletion by BAP FISH 1R1F but were positive for *CBFB-MYH11* fusion by RT-PCR (Table 3). Further investigation by targeted chromosomal sequencing identified two novel partner genes for *CBFB* rearrangement in cases #1 and #2 (data not included but will be published separately). The failure of RT-PCR for detection of *CBFB-MYH11* but not by concurrent FISH tests in case #4 was previously reported in two cases by Mrozek et al. [26]. The potential causes might be due to a rare or a novel *CBFB-MYH11* transcript other than *CBFB-MYH11* variants A, D, or E, and/or microdeletion or variation(s) affecting the target region of either *CBFB* and/or *MYH11* primers used for the RT-PCR that prevent detection of *CBFB-MYH11* transcript in this case. This case warrants further investigation using new approaches such as targeted RNA-Seq and/or WGS. One case (case #5) showed a normal karyotype and a normal BAP FISH result, but DF FISH revealed an insertion of *MYH11* into *CBFB* leading to *CBFB-MYH11* fusion, which was also confirmed by RT-PCR. Similar cases with cryptic *CBFB-MYH11* rearrangement were reported by Bidet et al. [10] and Douet-Guilbert et al. [11], but in their cases a part of *CBFB* was inserted into *MYH11*. In general, rearrangement caused by insertion is often cryptic by karyotyping and BAP FISH, unless the insertion is of a large size and/or unbalanced. Nevertheless, five cases also with atypical break-apart signal patterns by BAP FISH, e.g., 1R1F for *3′CBFB* deletion (*n* = 3), 1G1F for *5′CBFB* deletion (*n* = 1), and 1G2F for *5′CBFB* gain (*n* = 1) (Table 2 and Table 3), were RT-PCR negative as well. However, a *CBFB* rearrangement, likely with novel partner(s), similar to cases #1–#3 (Table 3), could not be entirely excluded in the rest of the cases. They also warrant a study by targeted RNA-Seq and/or WGS.

In this study, conventional cytogenetics analysis failed to detect a chromosomal aberration associated with *CBFB* rearrangement in about 4% of cases, due to no or insufficient metaphases for karyotype analysis, normal karyotype (cryptic), and/or complex rearrangements [25]. Occasionally, the inv(16)/t(16;16) abnormality could be easily overlooked during chromosomal analyses, especially when the chromosomal morphology is poor. Only after a subsequent positive FISH or RT-PCR result, retrospective assessment of the karyotype and especially with metaphase FISH images, a corrected report of karyotype results could be issued in these scenarios [5,23,27]. Interestingly, there were nine AML cases with a inv(16) (*n* = 2), t(16;16)(*n* = 2), t(16q22;v) (*n* = 2) (similar to cases #1–#3, Table 3), or add(16)(q22) (*n* = 3) that morphologically mimicked the classical inv(16)/t(16;16) but were negative by both BAP FISH and RT-PCR in this study (data not included). Therefore, inv(16)/t(16;16) with *CBFB-MYH11* fusion is not always appreciated by conventional cytogenetics. On the other hand, some other cases mimicking inv(16)/t(16;16) morphology may not have *CBFB-MYH11* fusion at all. FISH testing played an important or even a decisive role in the exclusion of inv(16)/t(16;16) AML in all these scenarios. A confirmation of *CBFB* rearrangement and consequently the diagnosis of inv(16)/t(16;16) AML are decisive for the clinical management of the patients, e.g., whether to administer cytarabine-based intensive chemotherapy and the likelihood of a favorable response [3,28,29,30].

Although inv(16)/t(16;16) AML is widely considered as one of the favorable-risk categories of AML, approximately half of the patients with inv(16)/t(16;16) AML are actually not cured at all [1]. The prognostic impacts of ACAs in inv(16)/t(16;16) AML remain controversial in the literature. For example, Han et al. [28] recently reported that trisomy 8 may indicate a good prognosis, whereas all other ACAs (or non-trisomy 8 ACAs) may imply a poorer prognosis in inv(16)/t(16;16) AML patients. However, earlier studies by other groups suggested that trisomy 22 but not trisomy 8 was associated with a better outcome [26,27] or that there was no association between ACAs and prognosis in relapsed inv(16)/t(16;16) AML [24]. Several studies including our previous study reported that a complex karyotype, especially structural ACAs, may strongly indicate an inferior overall survival in inv(16)/t(16;16) AML [29,31,32]. Given the fact that only a small portion of patients (18/271, 6.6%) exhibited either apparent (*n* = 9) or cryptic (*n* = 9) AC16As in this cohort of inv(16)/t(16;16) AML cases, *CBFB* rearrangement-causing inv(16) or translocations involving chromosomes 16 are mostly simple, balanced chromosomal aberrations without AC16As in the majority of all inv(16)/t(16;16) AML cases. Therefore, we postulated that *CBFB* rearrangement-causing inv(16) and translocations without apparent and/or cryptic AC16As can be considered as equivalent to a “numerical” but not a structural chromosomal abnormality while defining the status of complex karyotype. By following this new rule, at least one structural ACA and another numerical ACA are required to define a complex karyotype, which, in turn, did show negative prognostic impact in our previous study [32]. This hypothesis is also supported by Mosna et al. [33] and Han et al. [28], who suggested that a complex karyotype, if defined by the presence of >= 4 chromosomal abnormalities, is associated with adverse survival in patients with inv(16)/t(16;16) AML. Further investigation is needed to explore the association between ACAs, especially structural ACAs, and prognosis in this cohort. Additional information obtained by CBFB FISH tests can be applied to further reveal cryptic structural abnormalities in this cohort. For example, if we defined the 1R1G1F signal pattern by BAP FISH as “balanced” CBFB rearrangement and all other signal patterns with a confirmed *CBFB* rearrangement (metaphase FISH, DF FISH, and/or RT-PCR) as “unbalanced” *CBFB* rearrangement, implying for simultaneous gain or loss of a whole or part of *CBFB*, or in other words, additional chromosome 16 aberrations (AC16As), nine more cases with cryptic AC16As were identified in this cohort, including gain or deletion of *3′CBFB*, *5′CBFB* as well as additional *CBFB* rearrangements that were not appreciated by conventional cytogenetics and/or RT-PCR at all (data not included). Given the fact that only a small portion of patients (18/271, 6.6%) exhibited either apparent (*n* = 9) or cryptic (*n* = 9) AC16As in this cohort of inv(16)/t(16;16) AML cases, *CBFB* rearrangement-causing inv(16) or translocations involving chromosomes 16 are mostly simple, balanced chromosomal aberrations without AC16As in most inv(16)/t(16;16) AML cases. The impacts of these additional FISH findings on (re)definition of a complex karyotype, clinical presentation including BM morphological changes, gene mutation profile, response to chemotherapy, and outcome were investigated in this cohort as well. However, they were beyond the main scope of this study and will be reported separately. Although the performance of CBFB BAP FISH and CBFB-MYH11 DF probe sets were not systemically compared in this study, an analysis of their design and coverages (Figure 1) suggests that the CBFB BAP FISH is more likely for detecting small aberrations (e.g., 150 kb to 1 MB) involving *CBFB* and flanking region than the CBFB-MYH11 DF probe set.

To date, the next-generation sequencing (NGS)-based methods such as whole genome sequencing (WGS) [34,35], whole transcriptome sequencing (WTS) [36,37,38], and targeted RNA sequencing (RNA-Seq) [39,40,41], with the enormous power of precise detection of all known and even novel translocations and/or fusions simultaneously, have been implemented as a diagnostic tool for hematologic malignancies including inv(16)/t(16;16) AML. Interestingly, FISH assays including CBFB FISH have been applied either as a tool for the confirmation of novel fusions and/or copy number variants (CNVs) or solving the discrepancies between these new methods and karyotype analysis in these reports [34,35,37]. Although implementation of these NGS-based methods in clinical diagnosis of myeloid neoplasia is still at a stage of development and validation in our institute and none of the cases in this cohort was tested with any of these new methods yet, however, based on the biology of these methods and the parameters used in the published reports, we can postulate the results, as summarized in Table 5, of these methods were applied to all 271 cases with *CBFB* rearrangement in our study. These new methods will firmly detect the *CBFB* rearrangement and/or *CBFB-MYH11* fusion if applied to cases in this cohort, and the WGS will also detect underlying structural abnormalities (e.g., inv(16)/t(16;16), insertion, or t(16q22;v)) for *CBFB* rearrangement and all exiting ACAs including the apparent AC16As if they are at levels above the limit of detection of WGS for each aberration, while the WTS and targeted RNA-Seq, by theory, will detect *CBFB-MYH11* and other novel *CBFB* fusions but not the underlying structural abnormalities, not other structural abnormalities, and not any copy number aberrations (CNAs) if present. Certainly, the WTS and targeted RNA-Seq can also provide additional mutation information. Therefore, in our opinion, one or more cases with atypical CBFB FISH signal pattern are necessary to be included in the validation of a NGS-based method(s) to better validate and test the new method(s) prior to the clinical implementation. Nevertheless, karyotyping and FISH tests can provide additional information that is not detected by NGS-based methods and they still play important roles in the multidisciplinary diagnostics for AML in the era of precision and individualized medicine [42].

In summary, BAP FISH can provide a quick and accurate result for *CBFB* rearrangement in more than 99% of *CBFB* rearranged AML when it shows a typical signal pattern (1R1G1F). Atypical FISH result showing 3′*CBFB* deletion (1R1F) is often associated with an unbalanced *CBFB* rearrangement, especially when it is co-exists with inv(16), and a confirmatory assay (e.g., RT-PCR) is warranted. Sometimes, an atypical signal pattern may indicate AC16As with potential clinical implication, and cases with atypical CBFB FISH signal pattern should be included in the validation of a NGS-based method(s) for detection of translocations/gene fusions for clinical diagnosis.

## 5. Conclusions

In our study with 271 cases with confirmed *CBFB* rearrangement identified from over 1600 AML cases by CBFB FISH and CBFB-MYH11 RT-PCR, over 5% of them initially presented as challenging results, including discrepancy between FISH and RT-PCR tests and/or atypical FISH findings, which are mostly caused by additional chromosome 16 aberrations (AC16As). These AC16As are generally not appreciated by conventional cytogenetic analysis and are also overlooked by other methods including RT-PCR. They are predictively undetected by all NGS-based methods if following the currently published parameters. More importantly, AC16As lead to re-defining the concept of complex karyotype, risk stratification, and prognosis of patients with inv(16)/t(16;16) AML. Therefore, we confirmed that FISH testing is an important diagnostic tool for inv(16)/t(16;16) AML. Its performance compared with the NGS-based methods remains to be determined.

## Figures and Tables

**Table 1:** Summary of CBFB BAP FISH Results on 1629 patients.

Reported Results	Number of Patients (%)
**Positive for rearrangement**	**262 (16.1%)**
**Normal**	**1234 (75.7%)**
**Abnormal**	**133 (8.2%)**
Extra copy(ies)	34 * (2.1%)
Loss of one copy	84 (5.2%)
gain + loss	2 (0.1%)
3′CBFB deletion	11 (0.7%)
5′*CBFB* deletion	1 (0.1%)
5′*CBFB* gain **	1 (0.1%)
**Total**	**1629 (100%)**

* Thirty-one patients exhibited one to two extra copies of *CBFB* and two cases showed a small subclone (3% and 7%, respectively) with five copies of *CBFB* and one case with *CBFB* amplification in 3% of cells. ** A small subclone (9% comparing to 89% of blasts).

**Table 2:** Comparison of concurrent *CBFB* BAP FISH and CBFB-MYH11 RT-PCR results in 974 cases.

CBFB BAP FISH Results	*CBFB-MYH11* Fusion by RT-PCR		
	Tests Performed	Positive (%)	Negative (%)
**Positive**	**262**	**258 (98.5%)**	**4 (1.5%)**
**Normal**	**645**	**1 (0.2%)**	**644 (99.8%)**
**Abnormal**	**67**	**8 (11.9%)**	**59 (88.1%)**
Extra copy(ies)	15	0 (0%)	15 (100%)
Loss of one copy	37	0 (0%)	37 (100%)
gain + loss	2	0 (0%)	2 (100%)
3′CBFB deletion	11	8 (73%)	3 (27%)
5′CBFB deletion	1	0 (0%)	1 (100%)
5′*CBFB* gain	1	0 (0%)	1 (100%)
**Total**	**974**	**267**	**707**

**Table 3:** Summary of 17 representative cases with discordant CBFB BAP FISH and CBFB-MYH11 RT-PCR results (cases #1–#5) and cases with atypical FISH signals.

Case	Karyotype	CBFB BAP FISH	CBFB-MYH11 RT-PCR	Feature
1	46,XY,t(1;16)(q21;q22)[8]/46,XY[1 2]	ish t(1;16)(q21;q22)(3′CBFB+; 5′CBFB+)[2]. nuc ish(CBFBx2)(5′CBFB sep 3′CBFBx1)[62/200]	Neg	t(1;16) Discordant
2	50,XY,t(2;16)(q37;q22),t(3;16)(p21;p13),+8,+21,+22,+mar[5]/51,idem[cp2]/46,XY[13]	nuc ish(CBFBx2)(5′CBFB sep 3′CBFBx1)[40/200]	Neg	t(2;16) Discordant
3	46,XX,inv(3)(q21q26.2),del(6)(q21q27),t(16;19)(q22;q13.3),del(17)(p12)[17]/46,XX,inv(3)(q21q26.2),t(11;15)(q14;q26.3)[1]//46,XY[2]	nuc ish(CBFBx2)(5′CBFB sep 3′CBFBx1)[139/200]	Neg	t(16;19) Discordant
4	46,XX,inv(16)(p13.1q22)[10]/46,XX[10]	nuc ish(CBFBx2)(5′CBFB sep 3′CBFBx1)[134/200] *	Neg	Discordant
5	46,XY,inv(9)(p12q13)[20]	nuc ish(CBFBx2)l200}. Negative *	Pos	Discordant
6	47,XY,der(16)inv(16)(p13.1q22)del(16)(q22q34),+mar[2]/46, XY[18]	nuc ish (5′CBFBx2,3′CBFBx1)(5′CBFB con 3′CBFBx1)[104/200]	Pos	3′*CBFB* del
7	47,XY,inv(16)(p13.1q22),+22[20]	nuc ish(5′CBFBx2,3′CBFBx1)(5′CBFB con 3′CBFBx1)[20/200]	Pos	3′*CBFB* del
8	46,XX,der(16)inv(16)(p13.1q22)del(16)(q22)[19]/47,sl,+8[1]	nuc ish(5′CBFBx2,3′CBFBx1)(5′CBFB con 3′CBFBx1)[180/200]	Pos	3′*CBFB* del
9	46,XY,der(16)inv(16)(p13.1q22)del(16)(q22)[19]/46,XY[1]	ish der(16)inv(16)(p13.1)(5′CBFB+)q22(3′CBFB-)del(16)(q22)[2] nuc ish(5′CBFBx2,3′CBFBx1)(5′CBFB con 3′CBFBx1)[182/200]	Pos	3′*CBFB* del
10	46,XY,inv(16)(p13.1q22)[13]/46,XY	nuc ish(5′CBFBx2,3′CBFBx1)(5′CBFB con 3′CBFBx1)[146/200]	Pos	3′*CBFB* del
11	46,XX,inv(16)(p13.1q22)[9]/46,idem,+8,+22[3]/46,idem[cp2]/ 46,XX,t(2;22)(p13;q11.2),del(18)(q21.1q23),−22[1]/46,XX[5]	nuc ish(5′CBFBx2,3′CBFBx1)(5′CBFB con 3′CBFBx1)[189/200]	Pos	3′*CBFB* del
12	46,XY,der(16)del(16)(p13,1)inv(16)(p13.1q22)[20]	nuc ish(5′CBFBx2,3′CBFBx1)(5′CBFB con 3′CBFBx1)[175/200] *	Pos	3′*CBFB* del
13	46,XX,der(11)t(11;16)(p15;p13.1)inv(16)(p13.1q22),der(16)t(11;16)inv(16)del(16)(q22)[18]/46,XX[2]	nuc ish(5′CBFBx2,3′CBFBx1)(5′CBFB con 3′CBFBx1)[200]	Pos	3′*CBFB* del
14	46,XX,del(16)(q22)[15]/46,XX[4]	nuc ish(5′CBFBx2,3′CBFBx1)(5′CBFB con 3′CBFBx1)[190/200]	Neg	3′*CBFB* del
15	47,XY,+8[5]/47,idem,der(16)inv(16)(p13.3q13)del(16)(q22q22)[15]	nuc ish(5′CBFBx2,3′CBFBx1)(5′CBFB con 3′CBFBx1)[154/200] *	Neg	3′*CBFB* del
16	43~45,XY,add(1)(q21),−5,+6,−7,−8,add(11)(p15),add(16)(q22),add(18)(q21.1), del(20)(q11.2q13.3),+1~2mar[cp16]/46,XY[4]	nuc ish(5′CBFBx2,3′CBFBx1)(5′CBFB con 3′CBFBx1)[138/200]	Neg	3′*CBFB* del
17	43–46,XY,-2,del(3)(p21p25),del(5)(q13q33),add(7)(q36),+8,add(11)(p15), add(12)(p12),del(12)(p13),−15,der(16)del(16)(q11.2q22)add(16)(q22),−17,−19,+2mar[cp20]	nuc ish(5′CBFBx1,3′CBFBx2)(5′CBFB con 3′CBFBx1)[178/200]	Neg	5′*CBFB* del

* CBFB-MYH11 DF FISH was performed on cases #4, #5, #12, and #15. Case #4 exhibited a typical 1R1G2F signal pattern, consistent with inv(16); case #5 exhibited 1R2G1F, consistent with an insertion of *MYH11* into *CBFB;* case #12 showed 1R1G1F with fusion signal on 16q, consistent with inv(16) plus del(16p); case #15 exhibited 2R2G without *CBFB-MYH11* fusion.

**Table 4:** Correlation between chromosomal analysis and CBFB BAP FISH results in this study.

CBFB BAP FISH	Chromosomal Analysis			
	Normal chr16s	inv(16)	t(16;16)	Others
Positive (*n* = 254)	2	232	17	3 *
Normal (*n* = 1)	1	0	0	0
3′CBFB deletion (*n* = 8)	0	8	0	0
Total (*n* = 263)	3	240	17	3

* t(1;16) (*n* = 1), t(2;16) (*n* = 1), and t(16;19) (*n* = 1) (cases #1–#3 in Table 3), respectively.

**Table 5:** Estimated possibilities that the NGS-based methods may detect abnormalities in this cohort of 271 cases with *CBFB* rearrangement.

Conditions	# of Cases	WGS *	WTG **	Targeted RNA-Seq ***
*CBFB* rearrangement/fusion	271	yes	yes	yes
inv(16)	240	yes ^ǂ^	no	no
t(16;16)	17	yes ^ǂ^	no	no
insertion	1	yes	no	no
t(16q22;v)	3	yes	no	no
AC16As	18	9/18 ^ǂǂ^	no	no
Other ACAs	121	uncertain ^ǂǂǂ^	no	no

#: numbers; NGS: next-generation sequencing; WGS: whole genome sequencing; WTS: whole transcriptome sequencing; RNA-Seq: RNA sequencing. * Following the criteria published by Duncavage et al. [34] (e.g., CNAs > 5 Mb; and SV: >100 Kb); ** following the report by Stengel et al. [37]; *** providing the RNA-Seq platform is partner gene-unrestricted [40]; ^ǂ^ the WGS may be unable to distinguish inv(16) from t(16;16); ^ǂǂ^ all nine cases with cryptic AC16As in this cohort would have not been detected by WGS. ^ǂǂǂ^ Detection of ACAs by WGS would depend on the size of clone(s) with each ACA on each specimen.

## Data Availability

The data presented in this study are available on request from the corresponding author.
